# Vascular dysfunction and the age‐related decline in critical power

**DOI:** 10.1113/EP091571

**Published:** 2023-11-07

**Authors:** Abigail Dorff, Christy Bradford, Ashley Hunsaker, Jake Atkinson, Joshua Rhees, Olivia K. Leach, Jayson R. Gifford

**Affiliations:** ^1^ Department of Exercise Sciences Brigham Young University Provo Utah USA; ^2^ Program of Gerontology Brigham Young University Provo Utah USA

**Keywords:** ageing, endothelial function, exercise blood flow, exercise intolerance

## Abstract

Ageing results in lower exercise tolerance, manifested as decreased critical power (CP). We examined whether the age‐related decrease in CP occurs independently of changes in muscle mass and whether it is related to impaired vascular function. Ten older (63.1 ± 2.5 years) and 10 younger (24.4 ± 4.0 years) physically active volunteers participated. Physical activity was measured with accelerometry. Leg muscle mass was quantified with dual X‐ray absorptiometry. The CP and maximum power during a graded exercise test (*P*_GXT_) of single‐leg knee‐extension exercise were determined over the course of four visits. During a fifth visit, vascular function of the leg was assessed with passive leg movement (PLM) hyperaemia and leg blood flow and vascular conductance during knee‐extension exercise at 10 W, 20 W, slightly below CP (90% CP) and *P*_GXT_. Despite not differing in leg lean mass (*P* = 0.901) and physical activity (e.g., steps per day, *P* = 0.735), older subjects had ∼30% lower mass‐specific CP (old = 3.20 ± 0.94 W kg^−1^ vs. young = 4.60 ± 0.87 W kg^−1^; *P* < 0.001). The PLM‐induced hyperaemia and leg blood flow and/or conductance were blunted in the old at 20 W, 90% CP and *P*_GXT_ (*P* < 0.05). When normalized for leg muscle mass, CP was strongly correlated with PLM‐induced hyperaemia (*R* ^2^ = 0.52; *P* < 0.001) and vascular conductance during knee‐extension exercise at 20 W (*R* ^2^ = 0.34; *P* = 0.014) and 90% CP (*R* ^2^ = 0.39; *P* = 0.004). In conclusion, the age‐related decline in CP is not only an issue of muscle quantity, but also of impaired muscle quality that corresponds to impaired vascular function.

## Abstract (highlights)

- **What is the central question of this study?**
  Does the age‐related decrease in critical power occur independently of changes in muscle mass and is it related to impaired vascular function?
- **What is the main finding and its importance?**
  The age‐related reduction in exercise tolerance, assessed by critical power, occurs independently of loss of muscle mass and is related to vascular function and exercise blood flow. Blood flow during exercise slightly below critical power reaches very high steady‐state levels that do not differ significantly from the maximum blood flow achieved during exercise above critical power.

## 1 INTRODUCTION

Exercise tolerance decreases with age, such that activities of daily living (e.g., walking and climbing the stairs) become increasingly difficult (Taylor et al., 2023). Age‐related declines in critical power (CP) might contribute to the decline in exercise tolerance and physical function associated with ageing (Gifford & Collins, 2021). As the boundary that separates steady‐state and non‐steady‐state exercise (Jones et al., 2019), a decrease in CP could cause previously sustainable, submaximal exercise to elicit uncompensable metabolic conditions that inexorably approach maximal levels [e.g., maximal O_2_ uptake (${\dot{V}}_{O_{2}\max}$)]. Our group and several others have provided evidence indicating that CP decreases with age (Fulton et al., 2023; Gifford & Collins, 2021; Neder et al., 2000; Overend et al., 1992), but the cause of this decrease has not been investigated thoroughly. Development of a greater understanding of which factors are associated with the age‐related decrease in CP might lead to more targeted therapies and interventions to treat or prevent exercise intolerance with ageing.

Critical power is strongly influenced by the ability to resynthesize ATP aerobically, and oxygen delivery appears to be key in determining CP (Poole et al., 2021). For example, Kirby et al. (2021) demonstrated that, in contrast to the prompt establishment of steady‐state levels of muscle oxygenation that occurs during exercise below CP, exercise above CP results in an inexorable decrease in muscle oxygenation that continuous to decrease as long as the exercise is performed. Given that muscle oxygenation represents the balance between muscle oxygen delivery and muscle oxygen consumption, the continuous decrease in muscle oxygenation observed when exercising above CP is probably attributable to the inability of the cardiovascular system to keep pace with the elevated oxygen demand of the skeletal muscle mitochondria. Interventions that increase or decrease muscle oxygen delivery are known to affect CP (Broxterman et al., 2014; Goulding et al., 2020; Hammer et al., 2020a; Vanhatalo et al., 2010).

Given that there is a finite amount of cardiac output, the proper redistribution of blood flow by the resistance vasculature (e.g., arterioles) strongly influences muscle oxygen delivery and exercise tolerance (Gifford et al., 2020; Hanson et al., 2020; Joyner & Casey, 2015). Poole et al. (2021) hypothesized that impaired vascular function, especially at the level of the resistance vasculature, could conceivably lead to impaired muscle oxygen delivery and reduced CP. As evidenced by the diminished hyperaemic response to passive leg movement (PLM) (Groot et al., 2015; Hydren et al., 2019; Mortensen et al., 2012; Trinity et al., 2015) and intra‐arterial infusion of acetylcholine (Seals et al., 2011), ageing is associated with impaired endothelial function in the resistance vasculature.

It seems possible that the age‐related decline in CP is associated with concomitant changes in vascular function and exercise blood flow. Nevertheless, in addition to being sensitive to oxygen delivery, CP is also strongly influenced by muscle mass (Collins et al., 2022). Given that muscle mass often decreases with age (Distefano & Goodpaster, 2018), it is possible that the age‐related decline in CP is merely the result of decreased muscle quantity, not muscle quality. Therefore, the purpose of this study was to test the hypothesis that CP is reduced in older adults, even when considering any differences in muscle mass, and that the reduced CP of older adults is related to impaired vascular function and reduced exercise blood flow.

## 2 MATERIALS AND METHODS

### 2.1 Participants

The Brigham Young University (BYU) Institutional Review Board approved this study (IRB2021‐108) before recruitment occurred. Participants were recruited primarily around BYU campus. Each volunteer was informed of the purpose of the study, experimental procedures and potential risks associated with the study before written consent was obtained. Guidelines put forth in the *Declaration of Helsinki* were followed, except that the study was not pre‐registered on clinicaltrials.org.

As described in Table 1, 10 young adults (five female and five male; age, 24.4 ± 4 years) and 10 older adults (five female and five male; age, 63.1 ± 3 years) completed this study. All subjects reported being physically active, having exercised on 3–5 days per week for 30 min for at least the past 6 months. All subjects had no history of cardiovascular disease or heart problems, no history of metabolic disease, no current prescription medications and no history of smoking or illicit drug use. All young female adults were not pregnant (verified by urine‐based pregnancy test), and all older female adults reported being post‐menopausal.

**TABLE 1:** Subject characteristics. The statistics were determined with Student's unpaired *t*‐tests, with 10 subjects (five females and five male) in each group. Data are means ± SD.

Parameter	Old	Young	*P*‐value
Number of subjects (female, male)	10 (5, 5)	10 (5, 5)	–
Age, years	**63.1 ± 2.5**	**24.4 ± 4.0**	**<0.001**
Height, cm	174.6 ± 9.9	174.2 ± 6.0	0.904
Body mass, kg	86.5 ± 19.1	72.0 ± 11.1	0.053
Body mass index, kg m^−2^	**28.0 ± 3.3**	**23.4 ± 3.3**	**0.006**
Leg lean mass, kg	9.2 ± 2.4	8.9 ± 1.5	0.729
Leg fat mass, kg	**5.0 ± 1.7**	**3.5 ± 1.5**	**0.039**
Quadriceps mass, kg	2.5 ± 0.5	2.4 ± 0.5	0.836
Haemoglobin concentration in blood, g dL^−1^	15.4 ± 1.4	15.8 ± 1.5	0.735
Number of steps per day	13,001 ± 2464	13,527 ± 3214	0.702
Average time in sedentary activity, min day^−1^	1156 ± 61	1159 ± 74	0.937
Average time in light activity, min day^−1^	205 ± 54	187 ± 46	0.480
Average time in moderate‐to‐vigorous activity, min day^−1^	80 ± 24	94 ± 36	0.327

Bold values indicate *P*‐value < 0.05.

### 2.2 Procedures

This study involved five visits of ∼90–120 min each. Visit 1 included consenting, measuring body composition with dual emission X‐ray absorptiometry (DEXA), and familiarizing subjects with the knee‐extension exercise. Visits 2–4 involved further familiarization with the exercise and a series of tests to task failure to determine each subject's CP, work prime (*W*′) and maximum power reached during a graded exercise test (*P*_GXT_). Visit 5 involved measuring vascular function with PLM‐induced hyperaemia, and determining blood flow and blood pressure at various intensities of exercise below and above CP.

### 2.3 Visit 1: Subject characterization and familiarization

#### 2.3.1 Assessment of body composition

A DEXA scan was performed according to the manufacturer's recommendations (Lunar iDXA, GE Healthcare, Chicago, IL, USA). Software on the DEXA machine automatically calculated the percentage of body fat, total lean mass, total fat mass, leg lean mass and leg fat mass.

After the DEXA scan, subjects underwent thigh volume measurements. While lying supine, the circumference of the upper (around inguinal crease), middle and lower (immediately above the top of the patella) thigh was measured with a standard flexible tape measure. Skinfold thickness of the anterior mid‐thigh was also measured with standard skinfold calipers. These measurements were used to estimate quadriceps mass size as described by Layec et al. (2014).

#### 2.3.2 Familiarization with knee‐extension exercise

Subjects were then familiarized with single‐leg knee extension (e.g., kicking) on a custom‐made knee‐extension ergometer, with a magnetically braked cycle ergometer providing the resistance (Corival CPET; Lode, Groningen, The Netherlands). Following an initial introduction to the movement and several minutes of practice, the subject performed a graded exercise test (GXT), while maintaining kicking frequency at 80 r.p.m. This test consisted of a 3 min warm‐up at 10 W, followed by an increase of 3 W every 1 min until task failure. Task failure was defined as the point when the subject could no longer maintain a kicking frequency of 80 ± 5 r.p.m. for a consecutive 5 s, despite strong verbal encouragement. Once the subject had reached task failure, the wattage was lowered for a cool‐down. The greatest power output maintained for 60 s was identified as the GXT maximum power (i.e., *P*_GXT_). After a 20 min break, the subject performed a verification practice test. This test consisted of a 5 min warm‐up at 40% of their *P*_GXT_ and was followed by a 5 min break. Before starting the test, they performed another 3 min warm‐up at 40% of *P*_GXT_. At the end of the 3 min warm‐up, the wattage was increased to the *P*_GXT_ wattage, and the subject completed the knee‐extension exercise until task failure.

#### 2.3.3 Assessment of blood haemoglobin concentration

During the 20 min break between the two familiarization tests, a finger was pricked with a 2.0 mm lancet in order to measure haemoglobin content. Haemoglobin content was determined by using the HemoCue Hb 801 microcuvettes and analyser (HemoCue America, Brea, CA, USA). This was repeated two or three more times for a total of three or four readings from the same finger prick. The average of the readings was recorded as the subject's haemoglobin concentration.

#### 2.3.4 Assessment of physical activity

An Actigraph accelerometer (GT9X, ActiLife v.6.13.4; ActiGraph, Pensacola, FL, USA) was given to each subject. The subjects were instructed to wear the device on the non‐dominant wrist for 7–10 days. This allowed us to assess physical activity levels for young and old subjects. Step count and the number of minutes per day spent in light, moderate and vigorous exercise were calculated to match the activity levels between the younger and older groups. Activity thresholds were based upon the thresholds recommended by Montoye et al. (2020).

### 2.4 Visits 2–4: Determination of CP and *P*_GXT_

**FIGURE 1:**
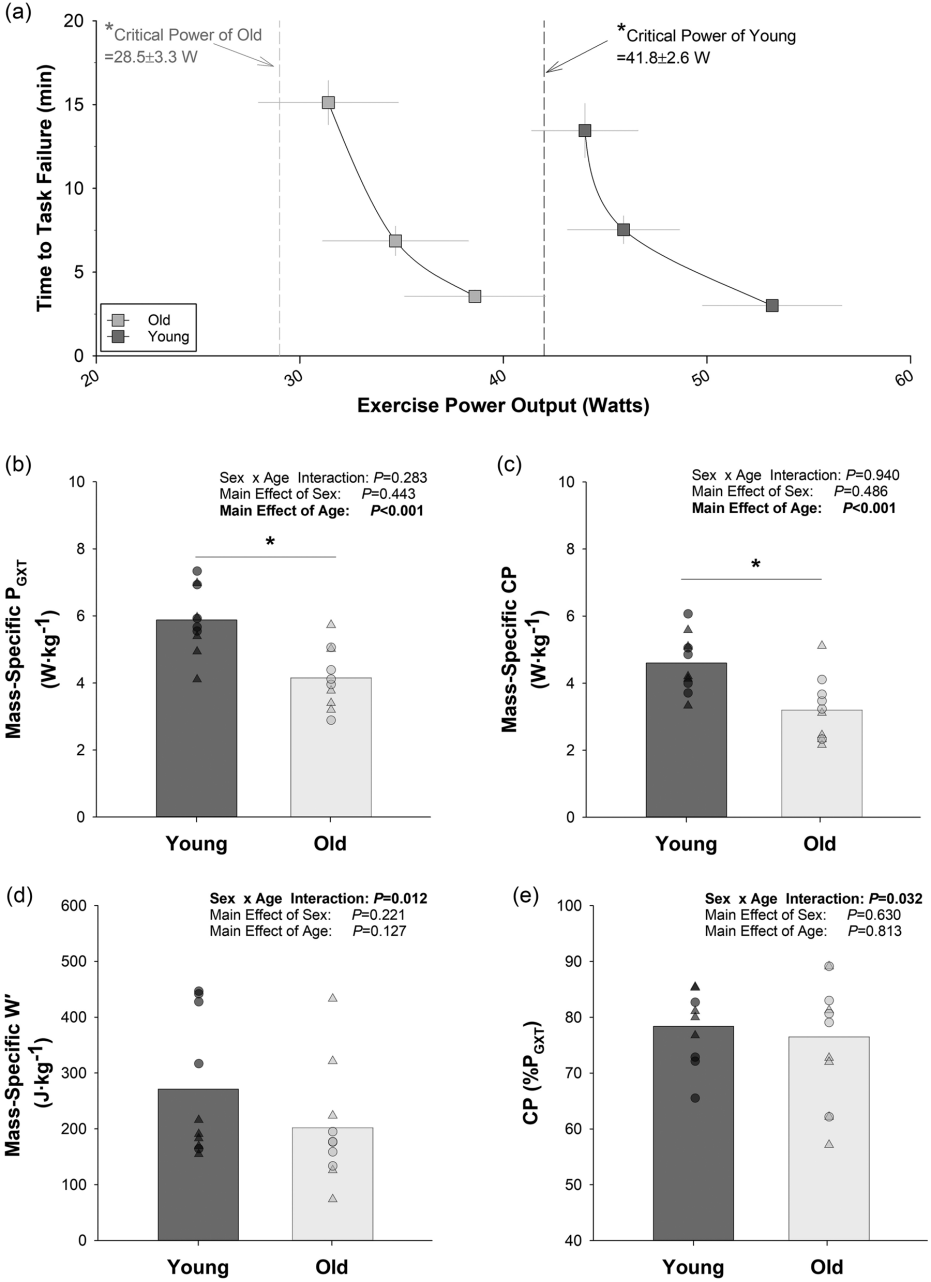
The effect of age on mass‐specific exercise tolerance during single‐leg knee‐extension exercise. (a) Illustration of the relationship between exercise power and time to task failure for young and older adults (data are means ± SEM). (b) Maximum power output achieved during a graded exercise test (*P*_GXT_) normalized by leg lean mass. (c) Critical power (CP) normalized by leg lean mass. (d) Work prime (*W*′) normalized by leg lean mass. (e) Critical power expressed as a percentage of *P*_GXT_. *Significant effect of age. Small symbols in (b–e) represent individual data points, with triangles representing females and circles representing males. Statistics were determined with a 2 × 2 ANOVA, with 10 subjects (five females and five male) in each group.

During visits 2–4, a series of constant‐power knee‐extension tests were performed until task failure to determine knee‐extension CP. The subjects started each test by warming up at 40% *P*_GXT_ for 5 min, followed by a 5 min rest period. Then each subject performed another warm‐up at 40% *P*_GXT_ for 3 min. Directly after the 3 min warm up, the resistance of the ergometer was set at 85% of the subject's *P*_GXT_. Subjects exercised at this intensity until task failure. Following a brief cool‐down and a 20 min rest period, the subjects then performed a second test to exhaustion at another power output between 80% and 105% of *P*_GXT_. Overall, three to five tests to task failure, ranging in duration between 2 and ∼15 min (Figure 1a), were completed over the course of three visits, with no more than two tests per visit. Twenty minutes after the final test to task failure, a final graded exercise test was performed to obtain a more accurate *P*_GXT_ compared with the familiarization day.

Critical power and *W*′ were both calculated with the linear model and the one‐over‐time model (Muniz‐Pumares et al., 2019). Estimates of CP and *W*′ from each model were not significantly different (CP, *P* = 0.123; W′, *P* = 0.187) and were strongly correlated with each other (CP, *R* ^2^ = 0.989, *P* < 0.001; *W*′, *R* ^2^ = 0.895, *P* < 0.001). Of the two models, the linear model provided the best fit (*R* ^2^ = 0.9973) and the lowest standard error for CP (SE = 2.9 ± 1.4%) and *W*′ (SE = 32.2 ± 70.7%) for most individuals. Therefore, we used exclusively data calculated via the linear model in all analyses presented in this paper.

### 2.5 Visit 5: Measurement of vascular function at rest and while exercising above and below CP

#### 2.5.1 Determination of resting vascular function

At least 48 h after the final determination of CP, subjects returned to the laboratory to have vascular function measured with the single passive‐leg movement test (Broxterman et al., 2017; Gifford & Richardson, 2017). Previous studies have demonstrated that the hyperaemic response to a single‐leg movement is nitric oxide dependent (Broxterman et al., 2017) and elicits a minimal chronotropic response (Venturelli et al., 2017). On arrival, subjects were seated in a raised chair, with their legs resting on a table slightly below their hip height. Subjects were connected to the Sun Tech Tango blood pressure machine (SunTech Tango+; SunTech Medical, Morrisville, NC, USA), in addition to a knee brace set to 90° of flexion and 180° of extension. The researcher then used the Doppler ultrasound machine (9L probe; General Electric Medical Systems, Milwaukee, WI, USA) to scan the subject's common femoral artery after the subject had been resting for ≥15 min. For the first 60 s, the blood velocity and artery diameter were recorded for a baseline. Next, a second researcher moved the right leg from 180° to 90° and then back a single time in 1 s. For the next 60 s, the researcher continued to record blood flow through the common femoral artery. This process was repeated three or four times, with a 5 min resting period between each test. Blood flow and vascular conductance were calculated as described below. Arterial diameter was calculated as the average of five diameter measurements assessed with software on the ultrasound machine at end diastole during baseline. Blood velocity data were binned into 1 s averages, and a 3 s rolling average was applied to smooth the data. The greatest blood flow achieved over 1 s was identified as the peak flow, and the total response was quantified as the area under the curve, as previously described (Gifford & Richardson, 2017). Resting blood pressure was determined as the average of three measurements performed while subjects sat recumbent during the 5 min break between tests.

#### 2.5.2 Determining the cardiovascular response to exercise at 10 and 20 W

Femoral artery blood flow and blood pressure were measured as subjects performed several bouts of knee‐extension exercise below and above CP. Initially, subjects performed knee‐extension exercise at 10 W for 5 min while femoral artery blood flow and brachial artery blood pressure were measured. After performing at 10 W for 5 min, the resistance on the ergometer was increased to 20 W. Subjects exercised at 20 W for 5 min or until task failure (whichever came first).

#### 2.5.3 Determining the cardiovascular response to exercise slightly below CP

We next investigated the blood flow response to exercise as near to CP as possible. Owing to the limitations of 1 W resolution on our cycle ergometer and error in the estimate of CP, 90% CP was the closest to CP that we could have everyone exercise, while still being certain that they did not exceed CP.

After a rest period of ≥10 min, subjects performed a warm‐up, exercising at 40% CP for 3 min, then immediately transitioned to an intensity of 90% CP. Subjects sustained this intensity for 10 min. During this test, a researcher scanned the femoral artery to record blood flow slightly below CP. Brachial artery blood pressure was taken every third minute as described above.

#### 2.5.4 Determining the cardiovascular response to exercise at *P*_GXT_

After another 10 min of rest, subjects exercised at 40% CP for 3 min, then immediately transitioned to an intensity of 100% *P*_GXT_ (determined during visit 4). Subjects maintained this intensity until task failure. Blood pressures were taken every third minute.

### 2.6 Data analysis and statistical analysis

#### 2.6.1 Calculation of femoral artery blood flow and conductance

Blood flow was measured with the Doppler ultrasound probe on the common femoral artery during the exercise. Specifically, a 9 MHz probe operating at a B‐mode frequency of 12 MHz and a Doppler frequency of 4 MHz was used to insonate the artery and measure blood velocity at an insonation angle of 60°. After data collection, blood flow was calculated with the following equation: Flow = mean blood velocity × π*r* ^2^, where mean blood velocity is the time‐averaged mean (TAmean) reported by the ultrasound machine and *r* is the radius of the artery, calculated as one‐half of the measured diameter. Arterial diameter was measured as the average of five measurements during each preceding baseline at a perpendicular angle along the central axis of the artery during end diastole using software on the ultrasound system (Gifford et al., 2016). Mean arterial pressure was calculated as diastolic blood pressure + ([systolic blood pressure − diastolic blood pressure]/3). Vascular conductance was calculated as the quotient of simultaneously measured blood flow and mean arterial pressure. For the purposes of this paper, exercise blood flow and conductance are represented as the average of the final 30 s of each stage, while peak flow and conductance measured during PLM are reported as the greatest 1 s average during the test (see above).

#### 2.6.2 Statistical analysis

The main variables of interest were CP, *W*′, peak blood flow during PLM, and blood flow, blood pressure and vascular conductance during submaximal exercise (10 W, 20 W and 90% CP) and maximal exercise (100% *P*_GXT_). Two‐way ANOVA, with age group (young vs. old) and sex (female vs. male), was performed to determine the difference in variables between young and old subjects. In the event of a significant *F*‐statistic, planned comparisons with Student's *t*‐tests were performed. Pearson correlation between the variables mentioned above was used to explore the relationship between the various indices of blood flow and exercise tolerance. The value of α was set at 0.05. Data are presented as the mean ± SD unless stated otherwise. Data are available upon request.

## 3 RESULTS

As described in Table 1, 20 active subjects (five young males, five older males, five young females and five older females) completed the full protocol for this study. Subjects were matched for activity level, measured by accelerometry (e.g., old = 13,001 ± 2464 steps per day vs. young = 13,527 ± 3214 steps per day; *P* = 0.702). Most subjects identified as Caucasian, while a few subjects (approximately) identified as being of Hispanic, African American, Asian, Pacific Islander or mixed descent.

### 3.1 Effect of age on exercise tolerance

#### 3.1.1 Effect of age on *P*_GXT_

A two‐way ANOVA indicated that absolute *P*_GXT_ was significantly different between the age groups (old = 36.2 ± 12.7 W vs. young = 52.1 ± 11.4 W; *P* = 0.003) and sexes (females = 36.8 ± 10.5 W vs. males = 53.5 ± 11.6 W; *P* < 0.001). No significant interaction was observed (*P* = 0.980). When *P*_GXT_ was normalized for body mass, a significant main effect of age was detected (old = 0.44 ± 0.10 W kg^−1^ vs. young = 0.73 ± 0.17 W kg^−1^; *P* < 0.001). As illustrated in Figure 1b, when normalized for leg lean mass, there was no longer a significant effect of sex (*P* = 0.443). Importantly, when normalized by leg lean mass, *P*_GXT_ was significantly lower in the older subjects (old = 4.15 ± 0.90 W kg^−1^ vs. young = 5.88 ± 0.99 W kg^−1^; *P* < 0.001), with no significant interaction observed (*P* = 0.283).

#### 3.1.2 Effect of age on critical power

When examining differences in CP with a two‐way ANOVA (age and sex), a significant main effect of age was observed, such that older individuals had a 32% lower absolute CP than younger individuals (Figure 1a; old = 28.5 ± W 10.4 vs. young = 41.8 ± 8.3 W; *P* = 0.007). A significant main effect of sex was also observed, such that females tended to have a lower absolute CP than males (females = 28.5 ± 10.4 W vs. males = 41.8 ± 8.3 W; *P* = 0.001). No interaction between age and sex was observed (*P* = 0.273). When normalized by total body mass, the main effect of age on CP was widened to a 40% difference between young (0.57 ± 0.13 W kg^−1^) and old (0.34 ± 0.10 W kg^−1^; *P* < 0.001). As illustrated in Figure 1c, the main effect of age persisted when normalized for leg lean mass, assessed by DEXA (old = 3.20 ± 0.94 W kg^−1^ vs. young = 4.60 ± 0.87 W kg^−1^; *P* < 0.001), and no sex differences (*P* = 0.486) or interactions (*P* = 0.940) were apparent.

#### 3.1.3 Effect of age on *W*′

When examining differences in CP with a two‐way ANOVA (age and sex), a significant interaction between age and sex for *W*′ existed (*P* = 0.038). Post hoc analysis revealed that young males exhibited a greater *W*′ than all other groups, such that there was a significant difference in *W*′ between young and old males (old males = 1897 ± 254 J vs. young males = 3535 ± 1445 J; *P* = 0.013), but not young and old females (old females = 1713 ± 774 J vs. young females = 1474 ± 294 J; *P* = 0.690). A significant main effect of sex was also observed (*P* = 0.016), such that, on average, males had a significantly greater *W*′ than females. As indicated in the interaction, this was primarily driven by the greater *W*′ of the young males. When *W*′ was normalized by total body mass, significant main effects of age (*P* = 0.029), sex (*P* = 0.044) and an age‐by‐sex interaction (*P* = 0.018) were detected. This interaction was driven primarily by a large decrease in *W*′ in old men (20.06 ± 4.37 J kg^−1^) compared with young men (46.97 ± 16.34 J kg^−1^; *P* = 0.016).

As illustrated in Figure 1d, the significant interaction between age and sex persisted when *W*′ was normalized for leg lean mass (Figure 1d; *P* = 0.012), such that young males exhibited a greater mass‐specific *W*′ than all other groups. Besides the interaction, no significant main effect of age (*P* = 0.127) or sex (*P* = 0.221) was observed for *W*′ when normalized by leg lean mass.

As illustrated in Figure 1e, a significant interaction was observed when considering CP as a percentage of *P*_GXT_ (*P* = 0.032), with the CP tending to occur at a higher percentage of *P*_GXT_ in older males than younger males (82.35 ± 1.23% vs. 75.03 ± 2.29%, respectively; *P* = 0.083), and at a lower percentage of *P*_GXT_ in older females than younger females (79.63 ± 3.96% vs. 81.72 ± 1.17%, respectively; *P* = 0.089). Notably, the percentage of *P*_GXT_ at which CP occurred was inversely related to mass‐specific *W*′ (*R* ^2^ = 0.52, *P* < 0.001).

### 3.2 Effect of age on blood flow when exercising near critical power

Older subjects reached significantly lower blood flows at 90% CP (old = 3293.37 ± 941.61 mL min^−1^ vs. young = 4197.57 ± 871.60 mL min^−1^; *P* = 0.035), and males had a greater absolute blood flow than females at 90% CP (males = 4299.00 ± 1019.05 mL min^−1^ vs. females = 3292.50 ± 72239 mL min^−1^; *P* = 0.020). No age‐by‐sex interaction was observed (*P* = 0.943). A significant main effect of age was also observed when examining maximum blood flow at *P*_GXT_ in absolute terms (young = 4305.36 ± 1377.69 mL min^−1^ vs. old = 3885.49 ± 944.45 mL min^−1^; *P* = 0.044), while no main effect of sex (*P* = 0.564) or age‐by‐sex interactions (*P* = 0.750) were observed.

**FIGURE 2:**
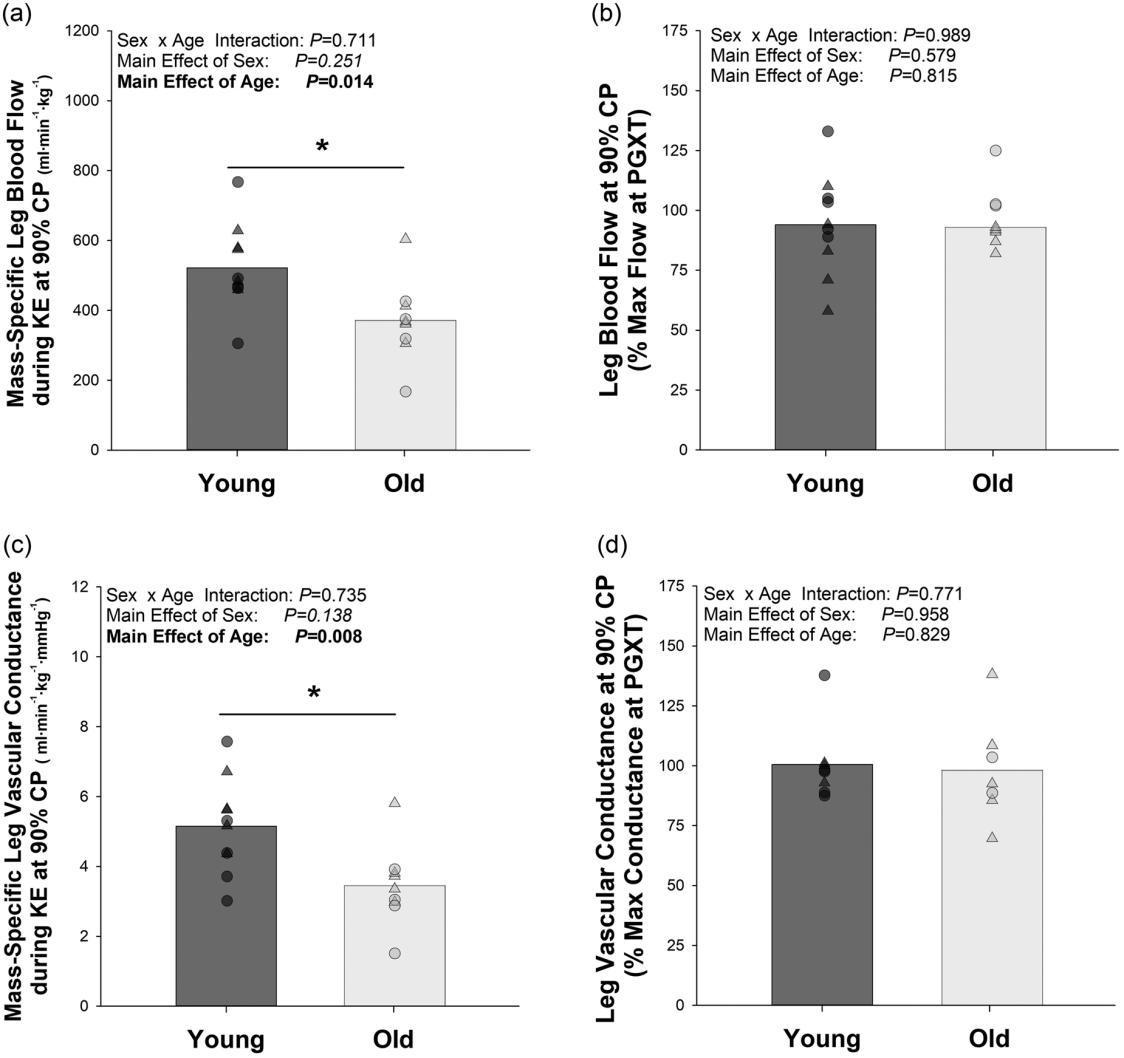
The effect of age on blood flow when exercising slightly below (90%) critical power (CP). (a) Leg blood flow, normalized by leg lean mass, during the 10th minute of exercise at 90% CP. (b) Blood flow during the 10th minute of exercise at 90% CP expressed as a percentage of maximum blood flow achieved when exercising at the maximum power achieved during a graded exercise test (*P*_GXT_). (c) Leg vascular conductance, normalized by leg lean mass, during the 10th minute of exercise at 90% CP. (d) Vascular conductance during the 10th minute of exercise at 90% CP expressed as a percentage of maximum conductance achieved when exercising at *P*_GXT_. *Significant effect of age. Small symbols represent individual data points, with triangles representing females and circles representing males. Statistics were determined with a 2 × 2 ANOVA, with 10 subjects (five females and five male) in each group for (a) and (c). Owing to difficulties in measuring blood flow/pressure during maximum exercise, only eight young (three female and five male) and seven old (five female and two male) subjects were included in the analysis for (b) and (d).

Figure 2a illustrates leg blood flow normalized by leg lean mass when exercising at 90% CP. When normalized by leg lean mass, sex differences in blood flow at 90% CP disappeared (*P* = 0.251), while the main effect of age persisted (young = 522.08 ± 123.47 mL min^−1^ kg^−1^ vs. old = 371.04 ± 115.84 mL min^−1^ kg^−1^; *P* = 0.014). No age‐by‐sex interaction was observed (*P* = 0.711). Blood flow achieved during the 10th minute of exercise at 90% CP did not differ significantly from maximum blood flow achieved when exercising at *P*_GXT_, averaging 93.56 ± 16.27% of maximum flow achieved during *P*_GXT_ (*P* = 0.122). As illustrated in Figure 2b, neither age (*P* = 0.815) nor sex (*P* = 0.579) affected the relationship between blood flow at 90% CP and *P*_GXT_.

As illustrated in Figure 2c, older individuals exhibited significantly lower vascular conductance per kilogram of leg lean mass than their young counterparts when exercising at 90% CP (*P* = 0.008). As illustrated in Figure 2d, neither age nor sex affected the relationship between conductance at 90% CP and *P*_GXT_. Notably, the vascular conductance achieved at 90% CP did not differ from maximum conductance (*P* = 0.897), averaging 99.38 ± 18.15% of the maximum vascular conductance achieved during *P*_GXT_. Absolute values for blood flow (i.e., not normalized by leg lean mass) are presented in Table 2.

**TABLE 2:** Absolute leg blood flow and vascular conductance during single‐leg knee‐extension exercise. Data presented here are absolute values, not normalized for leg lean mass. Abbreviations: CP, critical power; *P*_GXT_, maximum power achieved during a graded exercise test. Statistics were determined with Student's unpaired *t*‐tests, with 10 subjects (five females and five male) in each group. Data are means ± SD.

Parameter	Old	Young	*P*‐value
10 W			
Relative intensity of 10 W, % CP	**39 ± 20**	**26 ± 10**	**0.029**
Leg blood flow at 10 W, mL min^−1^	2307 ± 545	2408 ± 692	0.729
Leg conductance at 10 W, mL min^−1^ mmHg^−1^	25.7 ± 11.0	27.4 ± 8.1	0.708
20 W			
Relative intensity of 20 W, % CP	**78.5 ± 32**	**51.3 ± 11**	**0.029**
Leg blood flow at 20 W, mL min^−1^	**2688 ± 662**	**3377 ± 553**	**0.024**
Leg conductance at 20 W, mL min^−1^ mmHg^−1^	26.5 ± 11.0	35.2 ± 7.6	0.065
90% CP			
Absolute watts at 90% CP, W	**26.8 ± 11.0**	**35.5 ± 7.1**	**0.034**
Leg blood flow at 90% CP, mL min^−1^	**3260 ± 989**	**4524 ± 726**	**0.005**
Leg conductance at 90% CP, mL min^−1^ mmHg^−1^	**21.3 ± 9.6**	**33.6 ± 9.0**	**0.013**
100% *P*_GXT_			
Absolute watts at 100% *P*_GXT_, W	**38.2 ± 12.7**	**52.1 ± 11.4**	**0.019**
Leg blood flow at 100% *P*_GXT_, mL min^−1^	**3349 ± 966**	**4719 ± 992**	**0.009**
Leg conductance at 100% *P*_GXT_, mL min^−1^ mmHg^−1^	**29.3 ± 9.3**	**41.4 ± 9.2**	**0.011**

### 3.3 Effect of age on vascular function

#### 3.3.1 Effect of age on vascular conductance during exercise

**FIGURE 3:**
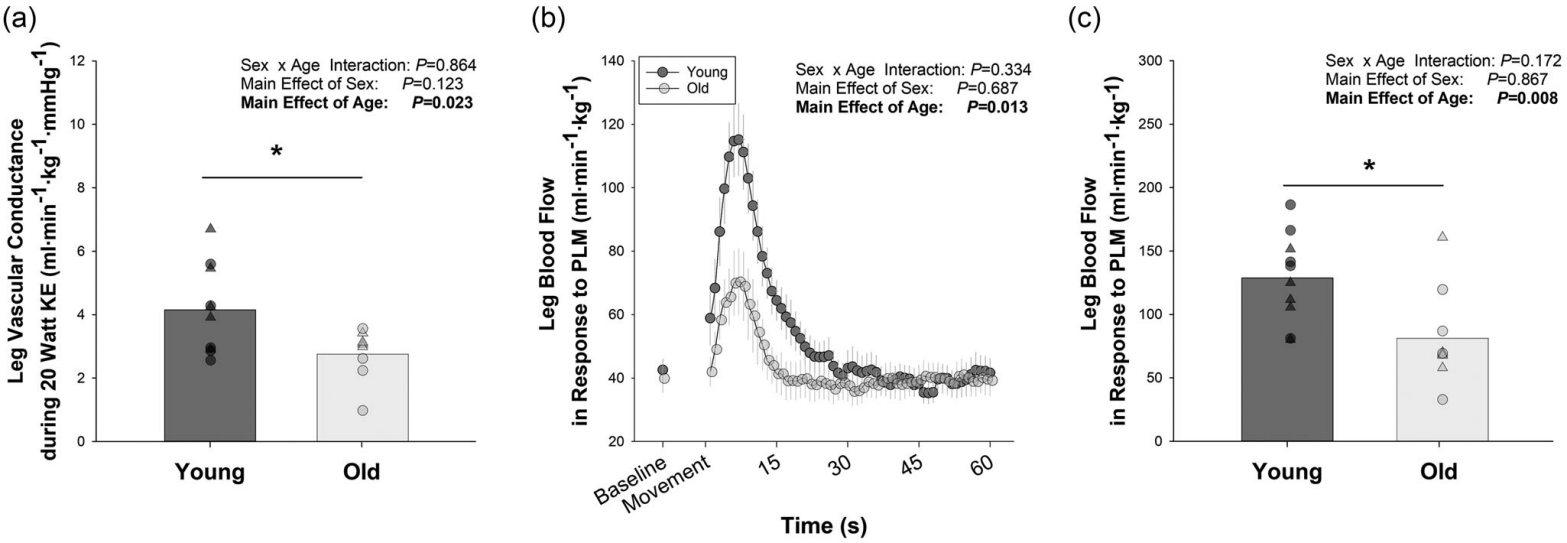
Effect of age on vascular function. (a) Leg vascular conductance when performing 20 W of knee‐extension (KE) exercise. (b) Total leg blood flow response to passive leg movement (PLM), normalized by leg lean mass, with statistics performed on the area under the curve. (c) Peak blood flow response to PLM. *Significant effect of age. Small symbols in (a) and (c) represent individual data points, with triangles representing females and circles representing males. Statistics were determined with a 2 × 2 ANOVA, with 10 subjects (five females and five male) in each group.

The vascular response to absolute power outputs of knee extension was quantified. No effect of age on conductance normalized in any fashion was apparent at 10 W (*P* > 0.250). At 20 W, older individuals exhibited a tendency for lower vascular conductance (young = 35.23 ± 7.6 mL min^−1^ mmHg^−1^ vs. old = 26.53 ± 10.99 mL min^−1^ mmHg^−1^; *P* = 0.080). As illustrated in Figure 3a, this comparison reached significance when normalized for leg lean mass (young = 4.15 ± 1.40 mL min^−1^ mmHg^−1^ kg^−1^ vs. old = 2.75 ± 0.83 mL min^−1^ mmHg^−1^ kg^−1^; *P* = 0.023). No effects of sex (*P* = 0.123) or sex‐by‐age interactions (*P* = 0.864) were observed in these comparisons.

#### 3.3.2 Effect of age on vascular endothelial function assessed during PLM

When considered in absolute terms, older adults exhibited significantly lower peak responses (*P* = 0.015) and total hyperaemic responses (*P* = 0.008) to PLM. A main effect of sex was observed for the peak response to PLM, such that females exhibited a significantly lower absolute response to PLM (*P* = 0.039).

Given that differences in muscle mass could mediate the observed differences in vascular function, we normalized the response to PLM by leg lean mass. As illustrated in Figure 3b, c, all sex differences disappeared, whereas all age differences persisted when normalizing for leg lean mass.

### 3.4 Relationship between vascular function, blood flow and exercise tolerance in young and old adults

**FIGURE 4:**
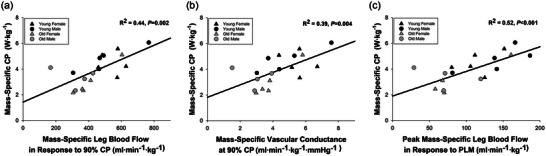
Relationship between critical power (CP) and vascular function. (a) The relationship between mass‐specific CP and mass‐specific leg blood flow during 90% CP knee‐extension exercise. (b) The relationship between mass‐specific CP and mass‐specific vascular conductance at 90% CP. (c) The relationship between mass‐specific CP and vascular function assessed by passive leg movement (PLM). Statistics were determined by linear regression, with five young females, five young males, five old females and five old males included in the analysis.

**FIGURE 5:**
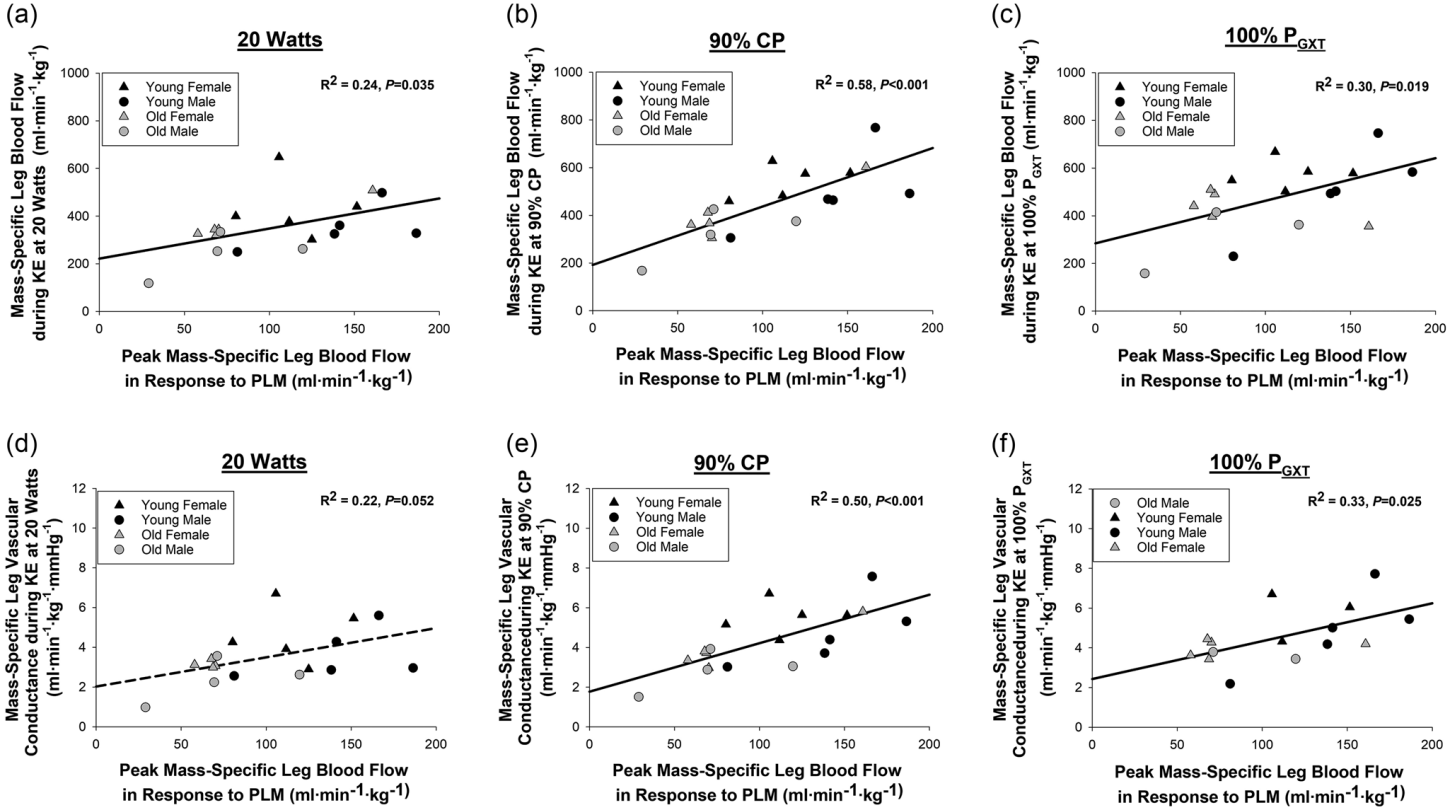
Relationship between resistance artery function and exercise blood flow and conductance. (a) The relationship between leg blood flow during 20 W knee‐extension exercise when normalized for muscle mass and peak leg blood flow in response to passive leg movement (PLM) when normalized for muscle mass. (b) The relationship between leg blood flow during 90% critical power (CP) knee‐extension exercise when normalized for muscle mass and peak leg blood flow in response to PLM when normalized for muscle mass. (c) The relationship between leg blood flow during maximum power achieved during a graded exercise test (*P*_GXT_) knee‐extension exercise when normalized for muscle mass and peak leg blood flow in response to PLM when normalized for muscle mass. (d) The relationship between leg vascular conductance during 20 W knee‐extension exercise when normalized for muscle mass and peak leg blood flow in response to PLM when normalized for muscle mass. (e) The relationship between leg vascular conductance during 90% CP knee‐extension exercise when normalized for muscle mass and peak leg blood flow in response to PLM when normalized for muscle mass. (f) The relationship between leg vascular conductance during *P*_GXT_ when normalized for muscle mass and peak leg blood flow in response to PLM when normalized for muscle mass. Statistics were determined by linear regression, with five young females, five young males, five old females and five old males included in the analysis, except for (c) and (f), which had only eight young (three female and five male) and seven old (five female and two male) subjects included in the analysis owing to difficulties in measuring blood flow/pressure during maximal exercise.

Absolute CP was correlated with peak leg blood flow in response to PLM (*R* ^2^ = 0.40; *P* = 0.004) and correlated with leg blood flow during knee‐extension exercise at 90% CP (*R* ^2^ = 0.30; *P* = 0.015). As illustrated in Figure 4, when normalizing for leg muscle mass, CP is strongly correlated with leg blood flow (*R* ^2^ = 0.44; *P* = 0.002; Figure 4a) and vascular conductance (*R* ^2^ = 0.39; *P* = 0.004; Figure 4b) during knee‐extension exercise at 90% CP and the peak hyperaemic response to PLM (*R* ^2^ = 0.52; *P* < 0.001; Figure 4c). As illustrated in Figure 5, the blood flow and vascular conductance responses to various intensities of knee‐extension exercise (e.g., 20 W, 90% CP and *P*_GXT_) were related to vascular endothelial function assessed by PLM.

## 4 DISCUSSION

The purpose of this study was to determine whether the age‐related decrease in CP occurs independently of changes in body composition and whether it is related to impaired muscle blood flow and vascular function. We observed that CP during knee‐extension exercise was lower in active older adults than in active young adults, even after controlling for muscle mass. We also observed that the age‐associated decrease in CP was strongly related to impaired blood flow and vascular conductance during exercise and to decreased endothelial function of the resistance vasculature. The context and implications of these findings is discussed below.

### 4.1 Does decreased muscle quality contribute to the age‐related reduction in CP?

As the boundary between steady‐state and non‐steady‐state exercise (Jones et al., 2019; Poole et al., 2016), a person's CP strongly influences the metabolic conditions, and consequently, the fatigue and sustainability that will be experienced at a given power output (Jones et al., 2008; Vanhatalo et al., 2016). Neder et al. (2000) previously showed that sedentary males >60 years old exhibited significantly lower absolute CP during cycling than sedentary males <30 years old. Gifford and Collins (2021) and Fulton et al. (2023) demonstrated that critical speed, the running surrogate of CP, decreases with age even in very active, world‐class masters’ athletes. Unfortunately, body composition was not assessed in these previous studies, making it unclear whether the reported decrease was merely attributable to age‐associated decreases in muscle mass or to increases in total body mass (Distefano & Goodpaster, 2018; Fleg et al., 2005).

In our study, we measured CP during single‐leg knee‐extension exercise. Consistent with previous studies (Fulton et al., 2023; Gifford & Collins, 2021; Neder et al., 2000), we observed that absolute CP during knee‐extension exercise was 32% lower in older adults than in young adults (Figure 1a). Importantly, CP was still 30% lower in the old adults when normalized for leg lean mass (Figure 1c). With age differences persisting after normalizing for muscle mass, it is clear that the age‐associated decrease in CP involves impairments in muscle quality, not only muscle quantity (i.e., mass). Importantly, typical age‐associated changes in muscle mass (e.g., sarcopenia) and body composition (e.g., increased body mass) are likely to compound the impact of the reduced muscle quality by requiring less muscle (of a lower quality) to lift more weight with each step (Distefano & Goodpaster, 2018; Fleg et al., 2005). Although indices of leg lean mass did not differ between groups in the present study, total body mass tended to be greater in the old adults (Table 1). Consequently, the disparity in CP between young and old adults grows to 40% when considering CP relative to total body mass (young = 0.57 ± 0.13 W kg^−1^ vs. old = 0.34 ± 0.10 W kg^−1^; *P* < 0.001). Thus, older adults, especially those who are overweight, are likely to be even more susceptible to fatigue during weight‐bearing exercises, such as walking, than non‐weight‐bearing exercises, such as knee extension or stationary cycling.

### 4.2 Does *W*′ vary with age?

The *W*′ can be defined as the amount of work and accompanying metabolic disturbance that can be tolerated when working above CP (Poole et al., 2016). Neder et al. (2000) showed that *W*′ was lower in sedentary males, as was CP. Likewise, Gifford and Collins (2021) reported that distance prime (*D*′; a surrogate of *W*′) was lower in 60‐year‐old athletes than in 35‐year‐old athletes. We observed a significant interaction between age and sex, such that mass‐specific *W*′ was lower in older males than young males, but there was no significant difference in *W*′ between young and older females, as illustrated in Figure 1d. In other words, male subjects exhibited a greater impact of age on *W*′ than female subjects. Additional studies designed to investigate sex differences are needed to confirm or reject these findings in our small sample; nevertheless, the differences in *W*′ between young males and young females are consistent with previous studies showing sex differences in *W*′ or *D*′ during cycling exercise (Collins et al., 2022; James et al., 2023) and running exercise (Gifford & Collins, 2021). Like CP, the deficit in *W*′ is exacerbated when considering total body mass, to the point that the younger subjects were able to perform ∼35% more work per kilogram than the older subjects (*P* = 0.028). Thus, even if young and old subjects had the same CP, the older subjects would reach exhaustion sooner than the younger subjects when performing weight‐bearing activities above CP.

The *W*′ appears to reflect the relationship between CP and *P*_GXT_, with a higher mass‐specific *W*′ being related to CP occurring at a lower percentage of *P*_GXT_ (*R* ^2^ = 0.52, *P* < 0.001). On average, young and old adults reached CP at the same percentage of *P*_GXT_ (Figure 1e); however, there was substantial variability from person to person, with CP occurring as low as 57% *P*_GXT_ and as high as 89% *P*_GXT_. This is in agreement with previous studies (Collins et al., 2022; James et al., 2023; Poole et al., 2016), demonstrating that CP cannot be estimated accurately as a fixed percentage (e.g., 80%) of *P*_GXT_ or ${\dot{V}}_{O_{2}\max}$.

Such variability in how CP relates to *P*_GXT_ might explain the inconsistency of responses to exercise between individuals, even when the exercise is performed at a given percentage of *P*_GXT_ or ${\dot{V}}_{O_{2}\max}$ (Collins et al., 2022, 2023; Meyler et al., 2021). For example, if a study tried to determine the impact of age on fatigability in our subjects by having them exercise at 80% *P*_GXT_, most of the young men would be exercising in the uncompensable, severe‐intensity domain, with its unique physiology, whereas most of the older men would be exercising in the compensable, heavy‐intensity domain, with its unique physiology (Poole et al., 2016). Thus, it might be more effective to describe and prescribe exercise in relationship to CP, not *P*_GXT_ or ${\dot{V}}_{O_{2}\max}$ power (Collins et al., 2022; Meyler et al., 2023; Poole & Jones, 2023).

### 4.3 Is leg blood flow reduced in older adults when exercising near CP?

Having observed that a decrease in muscle quality contributes to the age‐related decrease in CP, we next sought to determine whether decreases in the function of the vascular system might play a role. As illustrated in Figure 2, we measured leg blood flow and vascular conductance while young and old subjects exercised slightly below CP and again while they exercised to exhaustion at the maximum power previously achieved during a graded exercise test. Older subjects exhibited substantially lower blood flow (Figure 2a) and conductance (Figure 2c) per kilogram of lean mass than young subjects. Given that CP is considered a submaximal exercise, we were impressed at the high rates of leg blood flow achieved when exercising slightly below CP. Despite exercising at ∼30% lower power output (90% CP = 31.64 ± 2.39 W vs. 100% *P*_GXT_ = 45.15 ± 3.08 W; *P* < 0.001), the blood flow and conductance reached at 90% CP did not differ significantly from the maximum blood flow and conductance achieved during the *P*_GXT_ trial (Figure 2b, d). As suggested by Hammer et al. (2020b), CP appears to require maximal or nearly maximal rates of blood flow. This raises the possibility that, like ${\dot{V}}_{O_{2}\max}$ (Gifford et al., 2016; Levine, 2008), CP is strongly influenced, and potentially limited, by maximum blood flow.

**FIGURE 6:**
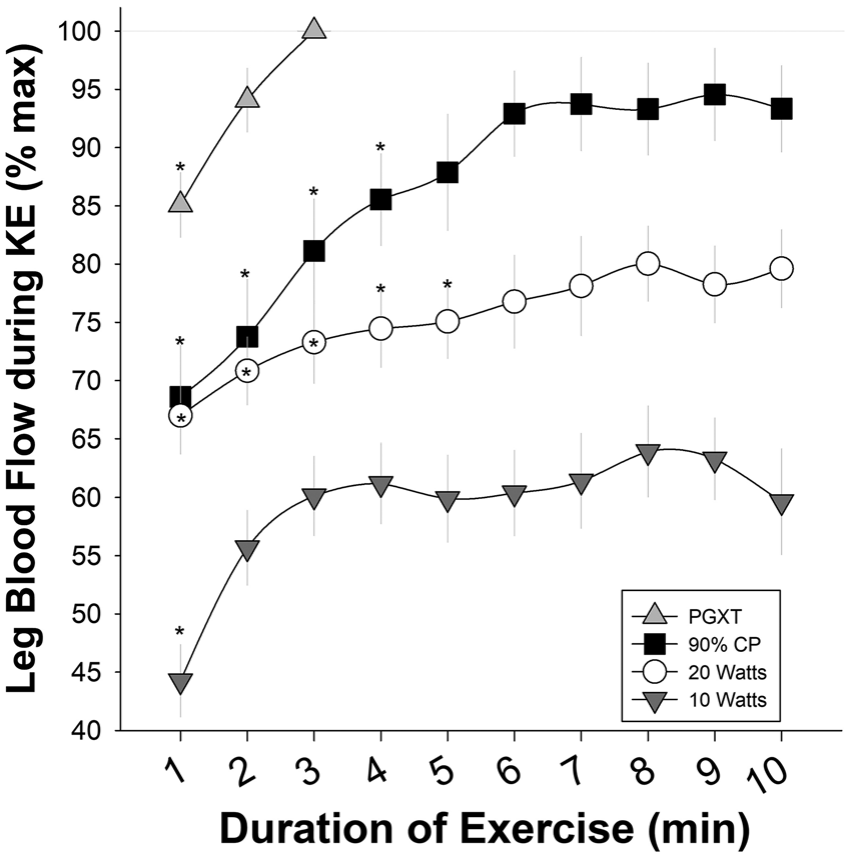
Leg blood flow response to knee‐extension (KE) exercise at four different intensities. All subjects performed 10 min of KE at 10 W, 20 W and 90% of their personal critical power (CP). Subjects also performed exercise at their previously determined maximum power (*P*_GXT_) until task failure. Leg blood flow is expressed as a percentage of maximum blood flow achieved when exercising at *P*_GXT_. Owing to missing time points (e.g., poor blood flow measurements at the onset of an exercise bout), only 15 subjects (nine young and six old) were included in this analysis. Data are not separated by age. Significant main effects of time (*P* < 0.001) were observed for each intensity. *Blood flow at that time point is significantly different from the final time point for that intensity. Data are presented as means ± SEM.

Our observation that blood flow at 90% CP reached near‐maximal levels might seem contradictory to observations by Copp et al. (2010), who reported substantially lower blood flow when rodents ran at ∼88% critical speed compared with ∼119% critical speed. We measured blood flow during 10 min of exercise at 90% CP, whereas Copp et al. (2010) measured flow ∼3.5 min into each exercise bout, probably before the slow component increase in flow (Figure 6) and O_2_ uptake fully matured. Therefore, it is very likely that flow and O_2_ uptake reached higher levels once a steady state was reached when exercising below critical speed.

### 4.4 Is the age‐associated reduction in CP related to vascular dysfunction?

Blood flow is determined primarily by the perfusion pressure across a vascular bed and the accumulative radius or size of the vascular bed (Joyner & Casey, 2015). The size of the vascular bed can be manipulated structurally, with angiogenesis and rarefaction, or it can be manipulated functionally, with vasodilatation and vasoconstriction (Joyner & Casey, 2015). Unfortunately, we were unable to measure capillary density in these subjects, and existing data are equivocal on whether capillary density decreases in active older adults (Landers‐Ramos & Prior, 2018). However, we did measure vasodilatory function of the leg with vascular conductance during KE exercise and with PLM‐induced hyperaemia (Figure 3).

As the quotient of flow and perfusion pressure, vascular conductance provides insight into the size and vasodilatory ability of the vascular bed. In addition to lower vascular conductance at 90% CP, older adults also exhibited significantly blunted conductance when exercising at the same absolute power output (20 W) as the young (Figure 3a). Importantly, mass‐specific conductance at 20 W (*R* ^2^ = 0.24, *P* = 0.039) and at 90% CP (*R* ^2^ = 0.39, *P* = 0.004; Figure 4a) were both significantly related to mass‐specific CP across all subjects, supporting the notion that CP might be related to vascular (dys)function (Poole et al., 2021).

Like others (Groot et al., 2015; Hydren et al., 2019; Mortensen et al., 2012; Trinity et al., 2015), we also observed a blunted hyperaemic response to a single passive movement of the leg. However, for the first time, we demonstrated that the age‐related difference in PLM‐induced hyperaemia persists even when normalizing for leg lean mass (Figure 3b, c), thereby providing further evidence of impaired muscle quality with ageing. Given that the hyperaemic response to PLM is strongly related to acetylcholine‐induced hyperaemia (Mortensen et al., 2012) and is mostly blunted with the inhibition of nitric oxide synthase (Broxterman et al., 2017; Mortensen et al., 2012), the blunted hyperaemic response of the old is suggestive of impaired endothelium‐dependent dilatation and reduced nitric oxide availability at the level of the resistance arteries that regulate blood flow (Broxterman et al., 2017; Gifford & Richardson, 2017; Limberg et al., 2020). Such impairments in dilatory function could conceivably reduce maximal muscle blood flow (Hanson et al., 2020) and contribute to decreased CP. In fact, PLM‐induced hyperaemia was strongly related to CP (Figure 4c) and the vascular response to exercise at 20 W, 90% CP and *P*_GXT_ (Figure 5).

In addition to causing limitations to oxygen delivery (Gifford et al., 2016), Poole et al. (2021) recently offered an additional hypothesis for how vascular dysfunction might impair CP. They suggested that impaired vascular function could result in an imbalance in oxygen delivery and consumption at the start of exercise, which would lower the intramuscular pressure of oxygen, leading to exaggerated metabolic stress and exercise intolerance. The strong relationship between PLM‐induced hyperaemia and CP (Figure 4c) supports this hypothesis put forward by Poole et al. (2021). Although these data do not establish a causal relationship between vascular function and CP, they do justify further investigation of the impact of vascular function on exercise tolerance. Research examining the impact of vasoactive interventions (Poole et al., 2021) on exercise tolerance seem especially justified by these data.

### 4.5 Experimental considerations

This study focused solely on healthy, unmedicated active adults. Although it seems likely that inactivity and diseases typical of ageing would exacerbate the discrepancies between young and old seen here, specific research on sedentary, diseased and medicated populations is needed to see whether this is the case. We also included equal representation of male and female subjects in this study. With our small sample size, sex‐difference data should be viewed as exploratory and interpreted with caution. Future studies are required to confirm or reject our exploratory findings.

The leg muscle mass used for normalization purposes was estimated with DEXA, which includes muscles other than the quadriceps femoris in its estimate. We also estimated quadriceps muscle mass with validated anthropometric measures (Layec et al., 2014). Like leg lean mass estimated by DEXA, estimated quadriceps mass did not differ between groups (Table 1). Importantly, leg lean mass obtained with DEXA and quadriceps mass obtained with anthropometry were significantly correlated (*r* = 0.66, *P* = 0.001). Given that normalizing the data by either estimate resulted in the same conclusions, we present only the data normalized by DEXA‐estimated leg lean mass.

We determined CP with two bouts of exhaustive exercise with 30 min of cool‐down, rest and warm‐up between them. We have found that this approach yields very reproducible measures of CP (coefficient of variation ≤ 3% across three separate sets of tests across a month) (James et al., 2023). Although previous data suggest that 30 min of recovery between trials results in valid estimates of CP (Karsten et al., 2017; Triska et al., 2021), it is possible that fatigue from the first bout affected the second bout, which might have caused us to overestimate or underestimate CP or *W*′. Exercise below CP is typified by steady‐state conditions (Jones et al., 2019; Poole et al., 2016). In Figure 6, we illustrate the leg blood flow response to the various exercise intensities for young and old adults combined. We tested for the existence of a steady state with post‐hoc analyses comparing blood flow at each time point with the final blood flow measured for that intensity. If we overestimated CP, a steady state would not be observed. Notably, exercise at 90% CP resulted in steady‐state blood flow after only 5 min, supporting the notion that the exercise was performed in the heavy domain. If we underestimated CP systematically, blood flow would probably be much lower than the maximum blood flow achieved during *P*_GXT_. Impressively, the data presented in Figures 2b and 6 indicate that steady‐state blood flow was reached at a very high percentage (∼94%) of the maximum flow observed during *P*_GXT_. With blood flow reaching such a high steady state that did not differ statistically from maximum blood flow (Figure 2b), it seems likely that, despite performing two exhaustive bouts in a day to determine CP, our estimates of CP were accurate. Nevertheless, it is still possible that the estimates of *W*′, which is often less reproducible (James et al., 2023; Karsten et al., 2017), were affected by our methodology and should be interpreted with caution.

Vascular function and exercise tolerance were assessed in a relatively small percentage of muscle mass that is not typically limited by cardiac output. Although the quadriceps are very relevant to locomotion, it is unclear how well the results of the present study apply to whole‐body exercise, such as walking, running or cycling. Given that blood flow at CP was not significantly different from maximum blood flow, it seems likely that CP could be sensitive to changes in maximum cardiac output, as was the case in a rodent model of heart failure (Craig et al., 2019).

## 5 CONCLUSIONS

Even when accounting for differences in muscle mass, physically active older adults exhibit reduced CP, indicating that changes in muscle quality contribute to the age‐related decline in exercise tolerance. Typical age‐related changes in body composition (e.g., decreased muscle mass and increased total body mass) are likely to magnify the exercise intolerance caused by impaired muscle quality, making previously sustainable activities of daily living very fatiguing. Differences in exercise blood flow and vascular function are strongly related to CP in young and old adults. Future research should determine whether improvements to vascular function have a meaningful impact on CP and exercise tolerance.

## AUTHOR CONTRIBUTIONS

This study was performed in the Human Performance Research Laboratory at Brigham Young University. Abigail Dorff conceived the work, acquired and analysed the data and drafted the manuscript. Christy Bradford acquired and analysed the data and critically revised the manuscript. Ashley Hunsaker acquired and analysed the data and critically revised the manuscript. Jake Atkinson acquired and analysed the data and critically revised the manuscript. Joshua Rhees acquired and analysed the data and critically revised the manuscript. Olivia Leach acquired and analysed the data and critically revised the manuscript. Jayson Gifford conceived the work, acquired and analysed the data and drafted the manuscript. All authors approved the final version of the manuscript and agree to be accountable for all aspects of the work in ensuring that questions related to the accuracy or integrity of any part of the work are appropriately investigated and resolved. All persons designated as authors qualify for authorship, and all those who qualify for authorship are listed.

## CONFLICT OF INTEREST

The authors declare no conflicts of interest.

## Data Availability

Data are available upon request to senior author.

## References

[eph13446-bib-0001] Broxterman, R. M. , Ade, C. J. , Wilcox, S. L. , Schlup, S. J. , Craig, J. C. , & Barstow, T. J. (2014). Influence of duty cycle on the power‐duration relationship: Observations and potential mechanisms. Respiratory Physiology & Neurobiology, 192, 102–111. 24361503 10.1016/j.resp.2013.11.010

[eph13446-bib-0002] Broxterman, R. M. , Trinity, J. D. , Gifford, J. R. , Kwon, O. S. , Kithas, A. C. , Hydren, J. R. , Nelson, A. D. , Morgan, D. E. , Jessop, J. E. , Bledsoe, A. D. , & Richardson, R. S. (2017). Single passive leg movement assessment of vascular function: Contribution of nitric oxide. Journal of Applied Physiology (1985), 123(6), 1468–1476. 10.1152/japplphysiol.00533.2017 PMC5814686 28860173

[eph13446-bib-0003] Collins, J. , Leach, O. , Dorff, A. , Linde, J. , Kofoed, J. , Sherman, M. , Proffit, M. , & Gifford, J. R. (2022). Critical power and work‐prime account for variability in endurance training adaptations not captured by V̇o(2max). Journal of Applied Physiology (1985), 133(4), 986–1000. 10.1152/japplphysiol.00344.2022 36107986

[eph13446-bib-0004] Copp, S. W. , Hirai, D. M. , Musch, T. I. , & Poole, D. C. (2010). Critical speed in the rat: Implications for hindlimb muscle blood flow distribution and fibre recruitment. The Journal of Physiology, 588(24), 5077–5087. 20962004 10.1113/jphysiol.2010.198382 PMC3036198

[eph13446-bib-0005] Craig, J. C. , Colburn, T. D. , Caldwell, J. T. , Hirai, D. M. , Tabuchi, A. , Baumfalk, D. R. , Behnke, B. J. , Ade, C. J. , Musch, T. I. , & Poole, D. C. (2019). Central and peripheral factors mechanistically linked to exercise intolerance in heart failure with reduced ejection fraction. American Journal of Physiology. Heart and Circulatory Physiology, 317(2), H434–H444. 31225988 10.1152/ajpheart.00164.2019 PMC7132310

[eph13446-bib-0006] Distefano, G. , & Goodpaster, B. H. (2018). Effects of Exercise and Aging on Skeletal Muscle. Cold Spring Harbor perspectives in medicine, 8(3), a029785. 28432116 10.1101/cshperspect.a029785 PMC5830901

[eph13446-bib-0007] Fleg, J. L. , Morrell, C. H. , Bos, A. G. , Brant, L. J. , Talbot, L. A. , Wright, J. G. , & Lakatta, E. G. (2005). Accelerated longitudinal decline of aerobic capacity in healthy older adults. Circulation, 112(5), 674–682. 16043637 10.1161/CIRCULATIONAHA.105.545459

[eph13446-bib-0008] Fulton, T. J. , Sundberg, C. W. , Arney, B. E. , & Hunter, S. K. (2023). Sex Differences in the Speed‐Duration Relationship of Elite Runners across the Lifespan. Medicine and Science in Sports and Exercise, 55(5), 911–919. 36728809 10.1249/MSS.0000000000003112 PMC10106388

[eph13446-bib-0009] Gifford, J. R. , & Collins, J. (2021). Critical speed throughout aging: Insight into the World Masters Championships. Medicine and Science in Sports and Exercise, 53(3), 524–533. 33560767 10.1249/MSS.0000000000002501

[eph13446-bib-0010] Gifford, J. R. , Garten, R. S. , Nelson, A. D. , Trinity, J. D. , Layec, G. , Witman, M. A. , Weavil, J. C. , Mangum, T. , Hart, C. , Etheredge, C. , Jessop, J. , Bledsoe, A. , Morgan, D. E. , Wray, D. W. , Rossman, M. J. , & Richardson, R. S. (2016). Symmorphosis and skeletal muscle ${\dot{V}}_{O_{2}\max}$: *In vivo* and *in vitro* measures reveal differing constraints in the exercise‐trained and untrained human. The Journal of Physiology, 594(6), 1741–1751. 26614395 10.1113/JP271229 PMC4799962

[eph13446-bib-0011] Gifford, J. R. , Hanson, B. E. , Proffit, M. , Wallace, T. , Kofoed, J. , Griffin, G. , & Hanson, M. (2020). Indices of leg resistance artery function are independently related to cycling ${\dot{V}}_{O_{2}\max}$ . Physiological Reports, 8(16), e14551. 32812353 10.14814/phy2.14551 PMC7435036

[eph13446-bib-0012] Gifford, J. R. , & Richardson, R. S. (2017). CORP: Ultrasound assessment of vascular function with the passive leg movement technique. Journal of Applied Physiology (1985), 123(6), 1708–1720. 10.1152/japplphysiol.00557.2017 PMC5814681 28883048

[eph13446-bib-0013] Goulding, R. P. , Roche, D. M. , & Marwood, S. (2020). Effect of hyperoxia on critical power and V˙O2 kinetics during upright cycling. Medicine and Science in Sports and Exercise, 52(5), 1041–1049. 31815830 10.1249/MSS.0000000000002234

[eph13446-bib-0014] Groot, H. J. , Rossman, M. J. , Trinity, J. D. , Layec, G. , Ives, S. J. , & Richardson, R. S. (2015). Passive leg movement‐induced vasodilation in women: The impact of age. American Journal of Physiology. Heart and Circulatory Physiology, 309(5), H995–H1002. 26188023 10.1152/ajpheart.00422.2015 PMC4591397

[eph13446-bib-0015] Hammer, S. M. , Alexander, A. M. , Didier, K. D. , & Barstow, T. J. (2020a). Influence of blood flow occlusion on muscular recruitment and fatigue during maximal‐effort small muscle‐mass exercise. The Journal of Physiology, 598(19), 4293–4306. 32721032 10.1113/JP279925

[eph13446-bib-0016] Hammer, S. M. , Alexander, A. M. , Didier, K. D. , Huckaby, L. M. , & Barstow, T. J. (2020b). Limb blood flow and muscle oxygenation responses during handgrip exercise above vs. below critical force. Microvascular Research, 131, 104002. 32198059 10.1016/j.mvr.2020.104002

[eph13446-bib-0017] Hanson, B. E. , Proffit, M. , & Gifford, J. R. (2020). Vascular function is related to blood flow during high‐intensity, but not low‐intensity, knee extension exercise. Journal of Applied Physiology (1985), 128(3), 698–708. 10.1152/japplphysiol.00671.2019 31917628

[eph13446-bib-0018] Hydren, J. R. , Broxterman, R. M. , Trinity, J. D. , Gifford, J. R. , Kwon, O. S. , Kithas, A. C. , & Richardson, R. S. (2019). Delineating the age‐related attenuation of vascular function: Evidence supporting the efficacy of the single passive leg movement as a screening tool. Journal of Applied Physiology (1985), 126(6), 1525–1532. 10.1152/japplphysiol.01084.2018 PMC6620662 30946637

[eph13446-bib-0019] James, J. J. , Leach, O. K. , Young, A. M. , Newman, A. N. , Mpongo, K. L. , Quirante, J. M. , Wardell, D. B. , Ahmadi, M. , & Gifford, J. R. (2023). The exercise power‐duration relationship is equally reproducible in eumenorrheic female and male humans. Journal of Applied Physiology (1985), 134(2), 230–241. 10.1152/japplphysiol.00416.2022 36548510

[eph13446-bib-0020] Jones, A. M. , Burnley, M. , Black, M. I. , Poole, D. C. , & Vanhatalo, A. (2019). The maximal metabolic steady state: Redefining the ‘gold standard.’. Physiological Reports, 7(10), e14098. 31124324 10.14814/phy2.14098 PMC6533178

[eph13446-bib-0021] Jones, A. M. , Wilkerson, D. P. , DiMenna, F. , Fulford, J. , & Poole, D. C. (2008). Muscle metabolic responses to exercise above and below the “critical power” assessed using 31P‐MRS. American Journal of Physiology. Regulatory, Integrative and Comparative Physiology, 294(2), R585–R593. 18056980 10.1152/ajpregu.00731.2007

[eph13446-bib-0022] Joyner, M. J. , & Casey, D. P. (2015). Regulation of increased blood flow (hyperemia) to muscles during exercise: A hierarchy of competing physiological needs. Physiological Reviews, 95(2), 549–601. 25834232 10.1152/physrev.00035.2013 PMC4551211

[eph13446-bib-0023] Karsten, B. , Hopker, J. , Jobson, S. A. , Baker, J. , Petrigna, L. , Klose, A. , & Beedie, C. (2017). Comparison of inter‐trial recovery times for the determination of critical power and W' in cycling. Journal of Sports Sciences, 35(14), 1420–1425. 27531664 10.1080/02640414.2016.1215500

[eph13446-bib-0024] Kirby, B. S. , Clark, D. A. , Bradley, E. M. , & Wilkins, B. W. (2021). The balance of muscle oxygen supply and demand reveals critical metabolic rate and predicts time to exhaustion. Journal of Applied Physiology (1985), 130(6), 1915–1927. 10.1152/japplphysiol.00058.2021 33914662

[eph13446-bib-0025] Landers‐Ramos, R. Q. , & Prior, S. J. (2018). The Microvasculature and Skeletal Muscle Health in Aging. Exercise and Sport Sciences Reviews, 46(3), 172–179. 29652695 10.1249/JES.0000000000000151 PMC6005745

[eph13446-bib-0026] Layec, G. , Venturelli, M. , Jeong, E. K. , & Richardson, R. S. (2014). The validity of anthropometric leg muscle volume estimation across a wide spectrum: From able‐bodied adults to individuals with a spinal cord injury. Journal of Applied Physiology (1985), 116(9), 1142–1147. 10.1152/japplphysiol.01120.2013 PMC4097823 24458749

[eph13446-bib-0027] Levine, B. D. (2008). VO2max: .. what do we know, and what do we still need to know? The Journal of Physiology, 586(1), 25–34. 18006574 10.1113/jphysiol.2007.147629 PMC2375567

[eph13446-bib-0028] Limberg, J. K. , Casey, D. P. , Trinity, J. D. , Nicholson, W. T. , Wray, D. W. , Tschakovsky, M. E. , Green, D. J. , Hellsten, Y. , Fadel, P. J. , Joyner, M. J. , & Padilla, J. (2020). Assessment of resistance vessel function in human skeletal muscle: Guidelines for experimental design, Doppler ultrasound, and pharmacology. American Journal of Physiology. Heart and Circulatory Physiology, 318(2), H301–H325. 31886718 10.1152/ajpheart.00649.2019 PMC7052621

[eph13446-bib-0029] Meyler, S. , Bottoms, L. , & Muniz‐Pumares, D. (2021). Biological and methodological factors affecting ${\dot{V}}_{O_{2}\max}$ response variability to endurance training and the influence of exercise intensity prescription. Experimental Physiology, 106(7), 1410–1424. 34036650 10.1113/EP089565

[eph13446-bib-0030] Meyler, S. , Bottoms, L. , Wellsted, D. , & Muniz‐Pumares, D. (2023). Variability in exercise tolerance and physiological responses to exercise prescribed relative to physiological thresholds and to maximum oxygen uptake. Experimental Physiology, 108(4), 581–594. 36710454 10.1113/EP090878 PMC10103872

[eph13446-bib-0031] Montoye, A. H. K. , Clevenger, K. A. , Pfeiffer, K. A. , Nelson, M. B. , Bock, J. M. , Imboden, M. T. , & Kaminsky, L. A. (2020). Development of cut‐points for determining activity intensity from a wrist‐worn ActiGraph accelerometer in free‐living adults. Journal of Sports Sciences, 38(22), 2569–2578. 32677510 10.1080/02640414.2020.1794244

[eph13446-bib-0032] Mortensen, S. P. , Askew, C. D. , Walker, M. , Nyberg, M. , & Hellsten, Y. (2012). The hyperaemic response to passive leg movement is dependent on nitric oxide: A new tool to evaluate endothelial nitric oxide function. The Journal of Physiology, 590(17), 4391–4400. 22733658 10.1113/jphysiol.2012.235952 PMC3473293

[eph13446-bib-0033] Muniz‐Pumares, D. , Karsten, B. , Triska, C. , & Glaister, M. (2019). Methodological Approaches and Related Challenges Associated With the Determination of Critical Power and Curvature Constant. Journal of Strength and Conditioning Research, 33(2), 584–596. 30531413 10.1519/JSC.0000000000002977

[eph13446-bib-0034] Neder, J. A. , Jones, P. W. , Nery, L. E. , & Whipp, B. J. (2000). The effect of age on the power/duration relationship and the intensity‐domain limits in sedentary men. European Journal of Applied Physiology, 82(4), 326–332. 10958376 10.1007/s004210000228

[eph13446-bib-0035] Overend, T. J. , Cunningham, D. A. , Paterson, D. H. , & Smith, W. D. (1992). Physiological responses of young and elderly men to prolonged exercise at critical power. European Journal of Applied Physiology and Occupational Physiology, 64(2), 187–193. 1555567 10.1007/BF00717959

[eph13446-bib-0036] Poole, D. C. , Behnke, B. J. , & Musch, T. I. (2021). The role of vascular function on exercise capacity in health and disease. The Journal of Physiology, 599(3), 889–910. 31977068 10.1113/JP278931 PMC7874303

[eph13446-bib-0037] Poole, D. C. , Burnley, M. , Vanhatalo, A. , Rossiter, H. B. , & Jones, A. M. (2016). Critical Power: An Important Fatigue Threshold in Exercise Physiology. Medicine and Science in Sports and Exercise, 48(11), 2320–2334. 27031742 10.1249/MSS.0000000000000939 PMC5070974

[eph13446-bib-0038] Poole, D. C. , & Jones, A. M. (2023). Critical power: A paradigm‐shift for benchmarking exercise testing and prescription. Experimental Physiology, 108(4), 539–540. 36719677 10.1113/EP091126 PMC10988433

[eph13446-bib-0039] Seals, D. R. , Jablonski, K. L. , & Donato, A. J. (2011). Aging and vascular endothelial function in humans. Clinical Science (London, England: 1979), 120(9), 357–375. 21244363 10.1042/CS20100476 PMC3482987

[eph13446-bib-0040] Taylor, J. A. , Greenhaff, P. L. , Bartlett, D. B. , Jackson, T. A. , Duggal, N. A. , & Lord, J. M. (2023). Multisystem physiological perspective of human frailty and its modulation by physical activity. Physiological Reviews, 103(2), 1137–1191. 36239451 10.1152/physrev.00037.2021 PMC9886361

[eph13446-bib-0041] Trinity, J. D. , Groot, H. J. , Layec, G. , Rossman, M. J. , Ives, S. J. , Morgan, D. E. , Gmelch, B. S. , Bledsoe, A. , & Richardson, R. S. (2015). Passive leg movement and nitric oxide‐mediated vascular function: The impact of age. American Journal of Physiology. Heart and Circulatory Physiology, 308(6), H672–H679. 25576629 10.1152/ajpheart.00806.2014 PMC4360052

[eph13446-bib-0042] Triska, C. , Hopker, J. , Wessner, B. , Reif, A. , Tschan, H. , & Karsten, B. (2021). A 30‐min rest protocol does not affect W', critical power, and systemic response. Medicine and Science in Sports and Exercise, 53(2), 404–412. 33416271 10.1249/MSS.0000000000002477

[eph13446-bib-0043] Vanhatalo, A. , Black, M. I. , DiMenna, F. J. , Blackwell, J. R. , Schmidt, J. F. , Thompson, C. , Wylie, L. J. , Mohr, M. , Bangsbo, J. , Krustrup, P. , & Jones, A. M. (2016). The mechanistic bases of the power‐time relationship: Muscle metabolic responses and relationships to muscle fibre type. The Journal of Physiology, 594(15), 4407–4423. 26940850 10.1113/JP271879 PMC4967754

[eph13446-bib-0044] Vanhatalo, A. , Fulford, J. , DiMenna, F. J. , & Jones, A. M. (2010). Influence of hyperoxia on muscle metabolic responses and the power‐duration relationship during severe‐intensity exercise in humans: A ^31^P magnetic resonance spectroscopy study. Experimental Physiology, 95(4), 528–540. 20028850 10.1113/expphysiol.2009.050500

[eph13446-bib-0045] Venturelli, M. , Layec, G. , Trinity, J. , Hart, C. R. , Broxterman, R. M. , & Richardson, R. S. (2017). Single passive leg movement‐induced hyperemia: A simple vascular function assessment without a chronotropic response. Journal of Applied Physiology (1985), 122(1), 28–37. 10.1152/japplphysiol.00806.2016 PMC5283853 27834672

